# Evaluation of Poly-Mechanistic Antiangiogenic Combinations to Enhance Cytotoxic Therapy Response in Pancreatic Cancer

**DOI:** 10.1371/journal.pone.0038477

**Published:** 2012-06-18

**Authors:** Niranjan Awasthi, Changhua Zhang, Winston Ruan, Margaret A. Schwarz, Roderich E. Schwarz

**Affiliations:** 1 Division of Surgical Oncology, Department of Surgery, The University of Texas Southwestern Medical Center, Dallas, Texas, United States of America; 2 Department of Pediatrics, The University of Texas Southwestern Medical Center, Dallas, Texas, United States of America; 3 Hamon Center for Therapeutic Oncology Research, Simmons Comprehensive Cancer Center, The University of Texas Southwestern Medical Center, Dallas, Texas, United States of America; University of Sheffield, United Kingdom

## Abstract

Gemcitabine (Gem) has limited clinical benefits in pancreatic ductal adenocarcinoma (PDAC). The present study investigated combinations of gemcitabine with antiangiogenic agents of various mechanisms for PDAC, including bevacizumab (Bev), sunitinib (Su) and EMAP II. Cell proliferation and protein expression were analyzed by WST-1 assay and Western blotting. In vivo experiments were performed via murine xenografts. Inhibition of in vitro proliferation of AsPC-1 PDAC cells by gemcitabine (10 µM), bevacizumab (1 mg/ml), sunitinib (10 µM) and EMAP (10 µM) was 35, 22, 81 and 6 percent; combination of gemcitabine with bevacizumab, sunitinib or EMAP had no additive effects. In endothelial HUVECs, gemcitabine, bevacizumab, sunitinib and EMAP caused 70, 41, 86 and 67 percent inhibition, while combination of gemcitabine with bevacizumab, sunitinib or EMAP had additive effects. In WI-38 fibroblasts, gemcitabine, bevacizumab, sunitinib and EMAP caused 79, 58, 80 and 29 percent inhibition, with additive effects in combination as well. Net in vivo tumor growth inhibition in gemcitabine, bevacizumab, sunitinib and EMAP monotherapy was 43, 38, 94 and 46 percent; dual combinations of Gem+Bev, Gem+Su and Gem+EMAP led to 69, 99 and 64 percent inhibition. Combinations of more than one antiangiogenic agent with gemcitabine were generally more effective but not superior to Gem+Su. Intratumoral proliferation, apoptosis and microvessel density findings correlated with tumor growth inhibition data. Median animal survival was increased by gemcitabine (26 days) but not by bevacizumab, sunitinib or EMAP monotherapy compared to controls (19 days). Gemcitabine combinations with bevacizumab, sunitinib or EMAP improved survival to similar extent (36 or 37 days). Combinations of gemcitabine with Bev+EMAP (43 days) or with Bev+Su+EMAP (46 days) led to the maximum survival benefit observed. Combination of antiangiogenic agents improves gemcitabine response, with sunitinib inducing the strongest effect. These findings demonstrate advantages of combining multi-targeting agents with standard gemcitabine therapy for PDAC.

## Introduction

Pancreatic ductal adenocarcinoma (PDAC) is one of the most aggressive human cancers and remains the fourth leading cause of cancer-related deaths in the United States. Rapid tumor progression, late diagnosis, early and aggressive metastasis and high resistance to conventional chemotherapy leads to exceptionally poor prognosis with a 5-year survival rate less than 5% [1]. Treatment of PDAC depends on the stage of the cancer; the overall resectability rate is only 10 to 15%, and postoperative recurrence is common [2], [3], [4]. Much attention has been focused towards systemic treatment options for PDAC for possible definitive or perioperative therapy benefit. Gemcitabine (Gem), a deoxycytidine nucleoside analog, is a cytotoxic agent that causes inhibition of DNA synthesis and cell death. The Food and Drug Administration (FDA) approved gemcitabine for the treatment of advanced PDAC in 1997. However, gemcitabine is clinically effective only in 20–30% of PDAC patients, leading to a median progression free survival of 5.7 months compared with 4.4 months in the 5-fluorouracil treated group [5]. Gemcitabine-based combination chemotherapy regimens have failed to show any meaningful survival advantage over single agent gemcitabine [6], [7]. These facts clearly demonstrate the urgent need for novel and more effective therapeutic strategies for PDAC.

Angiogenesis, a process by which tumors acquire blood supply for their continued growth, is essential for the progression of primary and metastatic solid tumors including PDAC. Angiogenesis is initiated by hypoxia, growth factors, cytokines, and activation of proto-oncogene and de-activation of tumor suppressor gene mechanisms [8]. Targeting angiogenesis to reduce tumor progression and metastasis may yield novel approach for combination therapy. Antiangiogenic agents such as anti-vascular endothelial growth factor (VEGF) agent bevacizumab (Bev), matrix metalloproteinase inhibitors (marimastat) and cyclooxygenase inhibitors (Celecoxib) have been studied in combination therapy in PDAC models with limited survival benefit [9], [10], [11]. Erlotinib, the epidermal growth factor receptor inhibitor, has to date been the only agent mediating a modest overall survival benefit in combination with gemcitabine [12].

**Table 1:** Percentage of cell viability in AsPC-1, HUVECs and WI-38 cells exposed to Gem, Bev, Su and EMAP, either alone or in combination.

Treatments and Combinations	AsPC-1				HUVECs				WI-38			
	Dose levels				Dose Levels				Dose levels			
	A	B	C	D	A	B	C	D	A	B	C	D
Gem alone	91±12	85±7	73±6	65±12	58±7	42±5	38±4	30±3	39±4	29±2	26±3	21±1
Bev alone	94±5	88±5	75±3	78±5	90±8	84±8	64±8	59±9	100±4	93±4	66±2	42±3
Su alone	100±9	94±6	78±6	19±4	47±8	44±6	38±1	14±7	109±8	99±1	83±2	20±2
EMAP alone	96±13	91±5	89±3	94±9	88±23	47±17	37±4	33±10	82±2	77±1	76±1	71±2
Gem+Bev	86±11	71±7	70±3	67±8	32±13	28±6	24±2	16±2	38±2	27±1	24±3	17±2
Gem+Su	79±5	59±6	55±1	19±4	43±13	31±2	29±1	8±5	39±3	28±1	21±1	16±3
Gem+EMAP	84±7	83±7	72±11	61±11	42±5	33±1	37±1	22±6	48±5	31±1	25±2	18±2
Bev+Su	80±16	64±2	57±3	22±1	64±8	49±6	35±3	18±2	81±10	73±6	46±1	21±2
Bev+EMAP	108±5	104±4	107±7	98±5	62±7	55±4	36±11	24±5	113±9	106±9	73±2	40±5
Su+EMAP	100±6	96±10	81±1	22±8	60±6	59±6	53±7	26±10	75±2	67±3	67±7	20±1
Gem+Bev+Su	83±5	75±4	59±4	27±3	54±13	38±5	32±7	20±4	34±1	24±3	20±1	17±3
Gem+Bev+EMAP	85±1	81±1	76±8	67±9	50±6	39±2	37±4	33±6	34±4	30±1	24±2	17±2
Gem+Su+EMAP	81±3	76±2	65±5	21±1	50±7	48±3	39±6	25±5	36±3	24±1	19±1	17±2
Bev+Su+EMAP	94±8	70±3	57±3	17±3	80±6	74±1	53±4	23±4	84±5	73±6	44±3	20±2
Gem+Bev+Su+EMAP	82±7	77±8	63±11	22±2	61±2	53±4	48±6	21±4	53±3	35±1	30±1	21±2

Cells were seeded into 96-well plates and treated with Gem, Bev, Su and EMAP. A represents 100 nM for Gem, Su, E and 1 µg/ml for Bev; B represents 500 nM for Gem, Su, E and 10 µg/ml for Bev; C represents 1 µM for Gem, Su, E and 100 µg/ml for Bev; D represents 10 µM for Gem, Su, E and 1000 µg/ml for Bev. After 72 hours incubation, 10 µl WST-1 reagent was added to each well, and absorbance of color produced was measured at 450 nm that correlates with the number of viable cells in the well. Data are expressed as the mean value ± standard deviation of quadruplicate determinants.

Many reports in the literature suggest that VEGF signaling plays an important role in PDAC progression [13], [14], [15], [16]. Therefore bevacizumab, a recombinant humanized monoclonal antibody against VEGF, was evaluated in phase II and phase III clinical trials. Although the bevacizumab and gemcitabine combination showed some promise in a phase II trial, no significant improvement was observed in subsequent phase III studies [17]. Sunitinib (Su) is a multi-target receptor tyrosine kinase (RTK) inhibitor with antiangiogenic and antitumor activities [18], [19], [20]. Sunitinib inhibits RTKs expressed by tumor cells that are involved in tumor cell proliferation and survival including stem cell factor receptor (c-KIT), Fms-related tyrosine kinase 3 (FLT3), the glial cell-line derived neurotrophic factor receptor (RET) and colony-stimulating factor type 1 receptor (CSF-1R) [18], [19]. Sunitinib also inhibits RTKs expressed on endothelial and mural cells, such as VEGF receptors (type 1 and 2) and platelet-derived growth factor (PDGF) receptors α and β [20], [21]. In human PDAC, VEGF receptors and PDGF receptors are over-expressed and have been correlated with poor prognosis [22], [23], [24]. Sunitinib has been shown to have antitumor efficacy in experimental PDAC [25], [26], [27]. Endothelial monocyte activating polypeptide II (EMAP) is a proinflammatory cytokine with antiangiogenic and antiendothelial activities. EMAP has potent effects on endothelial cells (ECs) such as inhibition of proliferation, migration and vascularization as well as induction of apoptosis [28], [29]. EMAP suppresses primary and metastatic tumor growth [28], [30], [31] that could be related to its ability to bind VEGF receptors and α5β1 integrin, leading to an interference in fibronectin- and VEGF signaling [32], [33]. EMAP has recently been shown to improve gemcitabine and docetaxel response in experimental PDAC [34], [35], [36]. The present study evaluated and compared combination treatment benefits of gemcitabine with three antiangiogenic agents bevacizumab, sunitinib and EMAP for potentially enhanced PDAC clinical applications.

**Figure 1:**
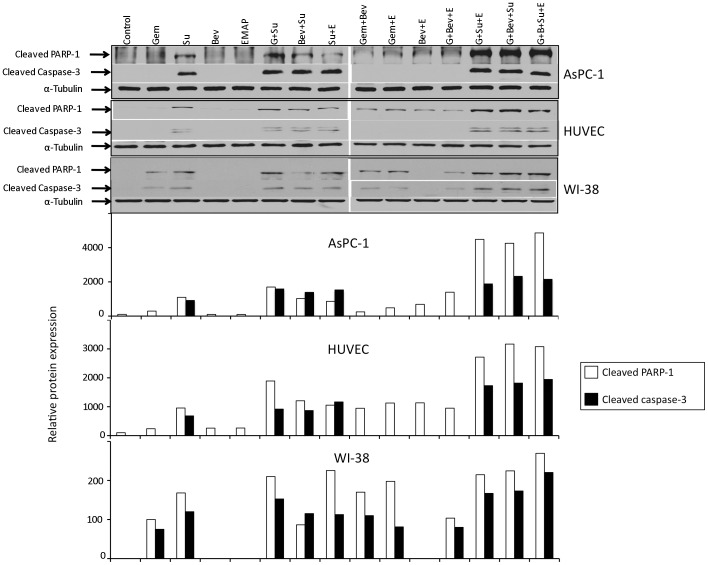
Evaluation of PARP-1 and caspase-3 cleavage. AsPC-1, HUVECs and WI-38 cell cultures were treated with Gem (10 µM), Bev (1 mg/ml), Su (10 µM) and EMAP (10 µM), either alone or in combination for 16 hours. Total cell lysate from treatment groups was subjected to SDS-PAGE and immunoblotting. Expression of α-tubulin was analyzed as internal loading control. Data are representative of two independent experiments with similar results.

## Materials and Methods

### Materials

Gemcitabine was purchased from Eli Lilly (Indianapolis, IN). Bevacizumab was purchased from Genentech (South San Francisco, CA). Sunitinib was purchased from LC Laboratories, Inc. (Woburn, MA). Recombinant human EMAP was prepared as previously described [37], while the cell proliferation reagent WST-1 was purchased from Roche Diagnostic Corporation (Indianapolis, IN).

### Cell Culture

The human pancreatic cancer cell line AsPC-1, human fibroblast cell line WI-38 and human umbilical vein endothelial cells (HUVECs) were all purchased from the American Type Culture Collection (ATCC, Rockville, MD). AsPC-1 and WI-38 cells were grown in RPMI 1640 medium and DMEM, respectively (Sigma Chemical Co. St. Louis, MO) supplemented with 10% fetal bovine serum (FBS). HUVECs were grown in EndoGRO-LS medium containing endothelial cell growth supplements (Millipore Corp., Billerica, MA).

### Cell Viability Assay

In vitro cell viability was evaluated by the WST-1 assay. Four thousand cells were plated in a 96-well plate and after 16 hours the medium was replaced with low serum containing medium. Cells were the treated with gemcitabine, bevacizumab, sunitinib and EMAP. The range of concentrations used for gemcitabine, sunitinib and EMAP (10 nM to 10 µM); and for bevacizumab (1 µg/ml to 1 mg/ml) was comparable to clinically achievable concentrations. After a 72-hour incubation, 10 µl WST-1 reagent was added in each well, and absorbance at 450 nm was measured after 2 hours using a microplate reader.

**Figure 2:**
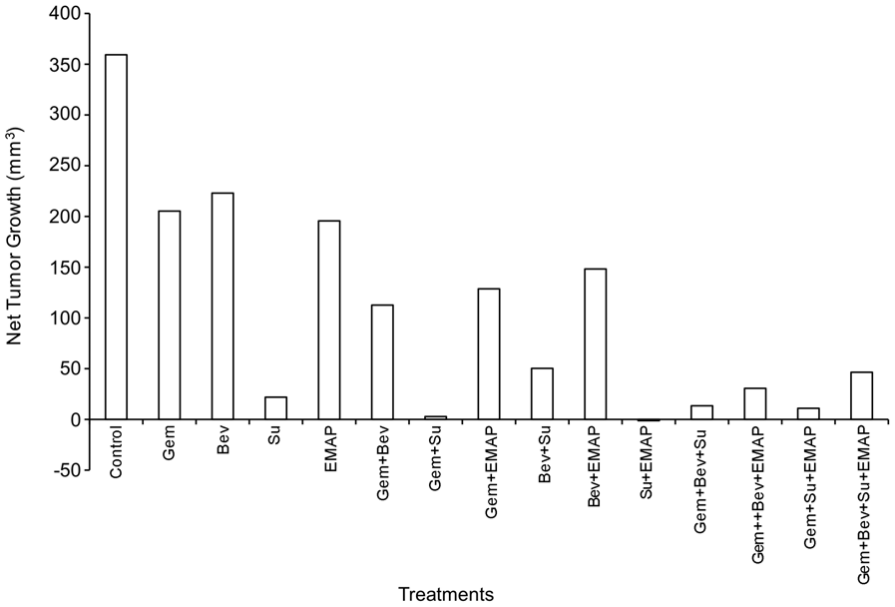
In vivo inhibition of local tumor growth. Nude mice were subcutaneously injected with AsPC-1 cell (0.75×10^6^) and treated with Gem, Bev, Su and EMAP, either alone or in combination, for 2 weeks. Tumor growth was measured 2 times a week using calipers, and net tumor growth was calculated by subtracting tumor volume on the first treatment day from that on the final day. Data are representative of mean values from 6–8 mice per group.

**Figure 3:**
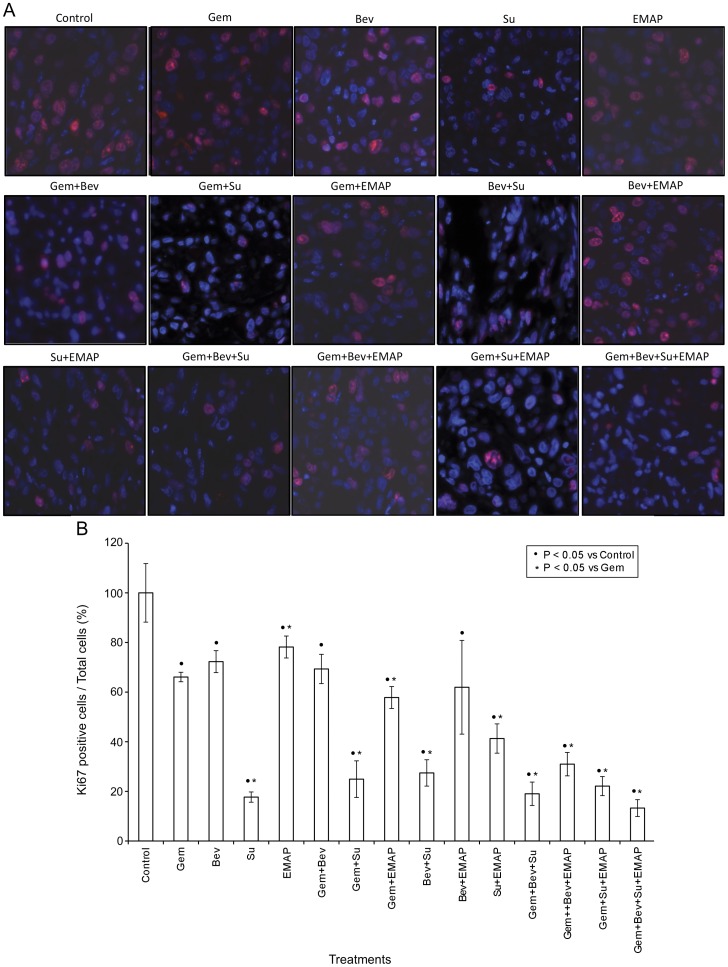
Measurement of intratumoral proliferative activity using Ki67 immunostaining. Nude mice were subcutaneously injected with AsPC-1 cells (0.75×10^6^) and treated with Gem, Bev, Su and EMAP, either alone or in combination, for 2 weeks. (A) Tumor tissue sections were immunostained with Ki67 nuclear antigen and photographed under a fluorescent microscope. (B) Ki67-positive cells were counted in five different high power fields. The data are expressed as the mean ± standard deviation. Symbols • and * represent significant differences (P<0.05) compared with controls and Gem group, respectively.

**Figure 4:**
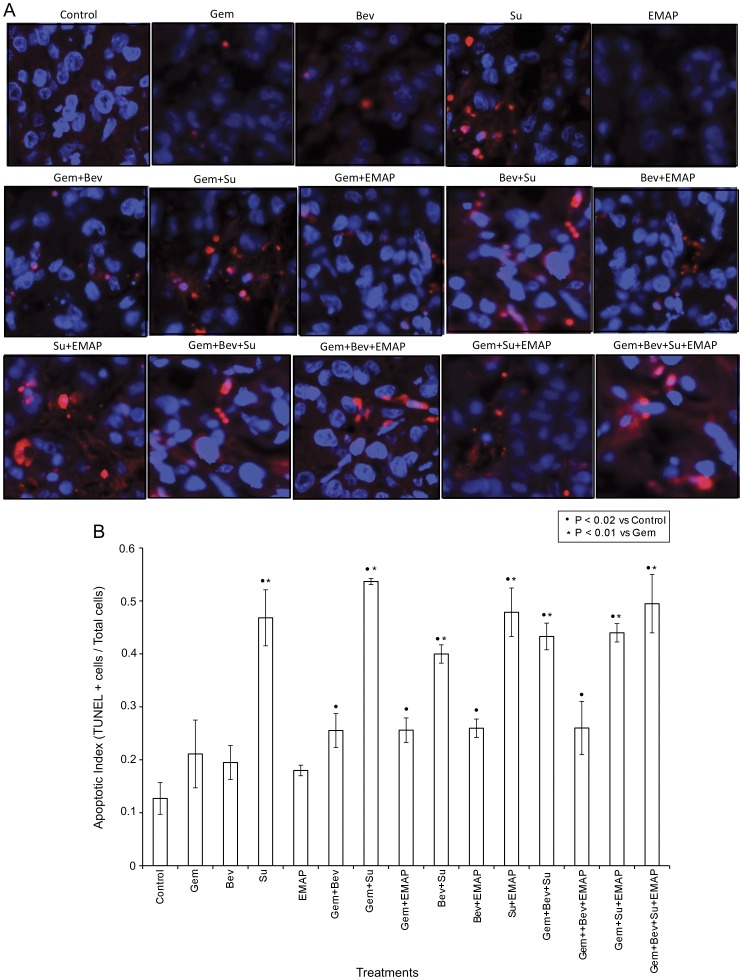
Measurement of intratumoral apoptotic activity using TUNEL staining. Nude mice were subcutaneously injected with AsPC-1 cells (0.75×10^6^) and treated with Gem, Bev, Su and EMAP, either alone or in combination, for 2 weeks. (A) Tumor tissue sections were stained with TUNEL procedure and photographed under a fluorescent microscope. (B) TUNEL-positive apoptotic cells were counted in five different high power fields. The data are expressed as the mean ± standard deviation. Symbols • and * represent significant differences (P<0.05) compared with controls and Gem group, respectively.

**Figure 5:**
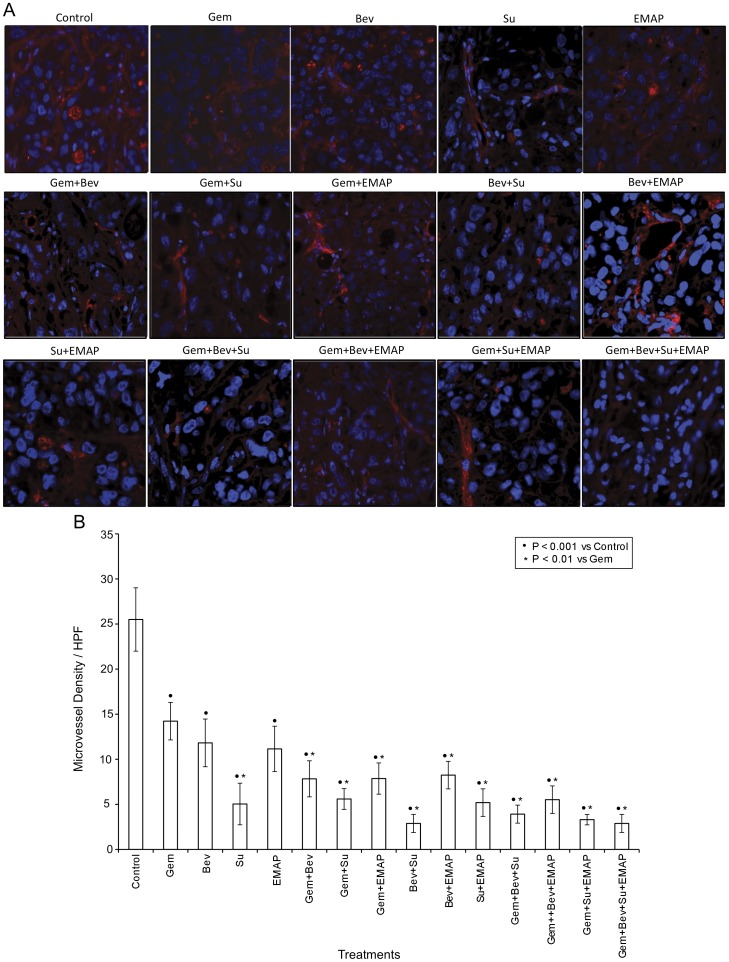
Evaluation of tumor vasculature by PECAM-1 immunostaining. Nude mice were subcutaneously injected with AsPC-1 cells (0.75×10^6^) and treated with Gem, Bev, Su and EMAP, either alone or in combination, for 2 weeks. (A) Tumor tissue sections were stained with PECAM-1 antibody and photographed under a fluorescent microscope. (B) PECAM-1 positive microvessel were counted in five different high power fields. The data are expressed as the mean ± standard deviation. Symbols • and * represent significant differences (P<0.05) compared with controls and Gem group, respectively.

**Figure 6:**
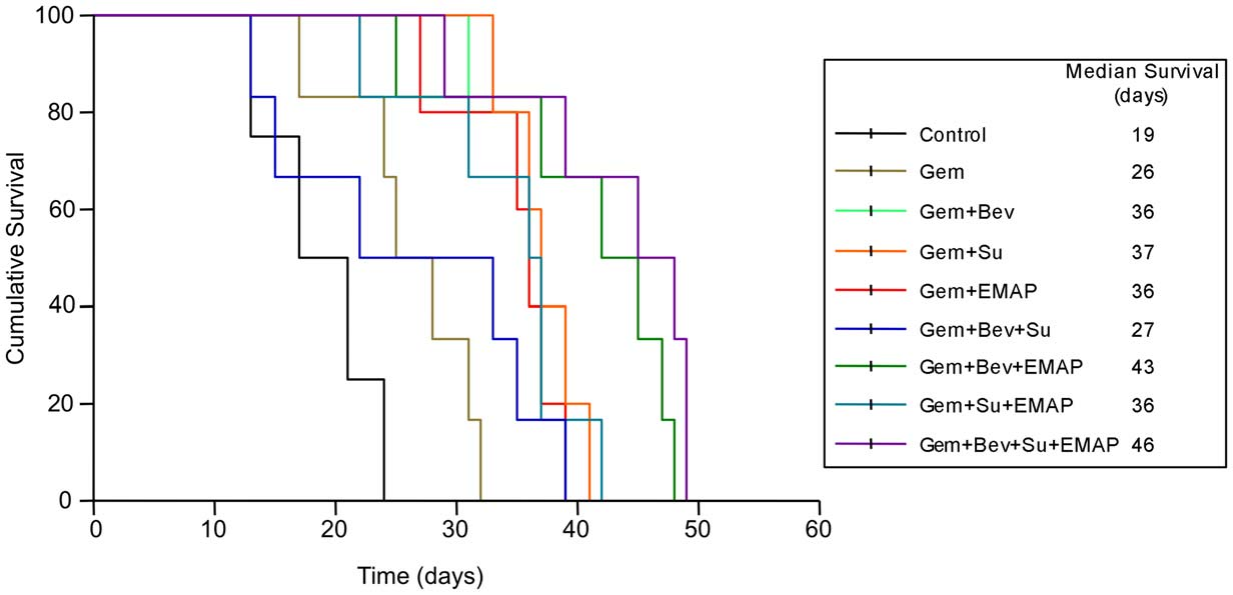
Animal survival time after treatment with Gem, Bev, Su and EMAP. AsPC-1 cells (0.75×10^6^) were intraperitoneally injected in SCID mice, and after 2 weeks followed by treatment with Gem, Bev, Su and EMAP, either alone or in combination, for a 2-week duration. The curve represents the animal survival time from the beginning of treatment.

### Western Blot Analysis

A sub-confluent cell monolayer was treated with gemcitabine (10 µM), bevacizumab (1 mg/ml), sunitinib (10 µM) or EMAP (10 µM) and incubated 12 hours for HUVECs and 24 hours for AsPC-1 and WI-38 cells. Total cell lysate was prepared, protein concentration measured and equal amounts of protein were separated by SDS-PAGE and transferred to PVDF membranes (Bio-Rad, Hercules, CA). The membranes were blocked for 1 hour in blocking solution (5% milk in TBS-T [Tris-buffered saline containing Tween-20]) and incubated overnight at 4°C with the following antibodies: cleaved poly (ADP-ribose) polymerase-1 (PARP-1), cleaved caspase-3 (Cell Signaling Technology, Beverly, MA) or α-tubulin (Sigma). The membranes were then incubated with corresponding HRP-conjugated secondary antibodies (Pierce Biotechnologies, Santa Cruz, CA) for 1 hour at room temperature. Specific bands were detected using the enhanced chemiluminescence reagent (ECL, Perkin Elmer Life Sciences, Boston, MA) on autoradiographic film and quantitated by densitometry.

### Tumor Implantation and in vivo Tumor Growth Experiment

All animal procedures and care were performed according to the guidelines and approved protocols of the University of Texas Southwestern Medical Center (Dallas, TX) Institutional Animal Care and Use Committee (Animal Protocol Number 2008-0348). Athymic female nude mice (aged 4–8 weeks) were used in subcutaneous xenograft model. Human pancreatic cancer AsPC-1 cells (0.75×10^6^) were subcutaneously injected in each mouse. After 14 days when all mice had measurable tumor, mice were randomly grouped (n = 6 to 8 per group) and treated intraperitoneally with PBS (control), gemcitabine (100 mg/kg, twice weekly), bevacizumab (10 µg per mouse, twice weekly), sunitinib (40 mg/kg for 1^st^ week, 20 mg/kg for 2^nd^ week, 5 times weekly) and EMAP (80 µg/kg, 5 times weekly) for 2 weeks. The tumor size in all mice was measured twice weekly by caliper. Tumor volume (V) was calculated by using the formula [V = ½ (L×(W)^2^], where L = length and W = width. Net growth in tumor size was calculated for each animal by subtracting tumor volume on the first day of treatment from that on the last day. After completion of treatment, all animals were euthanized, tumors were removed, weighed, dissected and processed for histological or immunohistochemical analysis.

### Histology and Immunohistochemical Analysis

Tumor tissue specimens fixed in 4% paraformaldehyde and embedded in paraffin were used for histological and immunohistological analysis. Intratumoral proliferative activity was measured using by Ki67 nuclear antigen staining as per manufacturer’s protocol (Abcam, Cambridge, MA). Briefly, paraffin-embedded tumor tissue sections were cut, deparaffinized, rehydrated and antigen retrieved. The tissue sections were incubated with CAS blocking buffer followed by 1-hour incubation with Ki67 antibody (1∶200 dilution). The tissue sections were then incubated with Cy3 (1∶200 dilution) secondary antibody (Jackson ImmunoResearch Laboratories, West Grove, PA) for 40 minutes. Slides were mounted using mounting solution containing 4′,6-diamidino-2-phenylindole (DAPI) (Invitrogen, Carlsbad, CA). Proliferative activity was evaluated by calculating Ki67-positive cells from five different high-power fields (HPF) in a blinded manner. For detecting microvessel density (MVD), tissue sections were incubated with 1∶100 dilution of PECAM-1 (CD-31) antibody (BD Pharmingen, Bedford, MA) overnight at 4°C. The tissue sections were then incubated with 1∶200 dilution of Cy3 secondary antibody for 40 minutes. Slides were mounted using mounting solution containing DAPI, and MVD was evaluated by counting PECAM-1 positive vessels within a microscopic HPF in a blinded manner. Intratumoral apoptosis was analyzed by staining tissue sections with “Apoptag Apoptosis Detection Kit” according to the manufacturer’s (Millipore) instructions. Fluorescence microscopy was used to detect fluorescent signals using IX81 Olympus microscope and images were captured with a Hamamatsu Orca digital camera (Hamamatsu Corporation, Bridgewater, NJ) with a DSU spinning confocal unit using Slidebook software (Intelligent Imaging Innovations, Philadelphia, PA).

### Animal Survival Analysis

Animal survival studies were performed using 6- to 8-week-old female SCID mice [38]. AsPC-1 (0.75×10^6^) cells were injected intraperitoneally in each mouse and after two weeks mice were randomly grouped (n = 6 to 8 per group) and treated intraperitoneally with PBS (control), gemcitabine (100 mg/kg, twice weekly), bevacizumab (10 µg per mouse, twice weekly), sunitinib (40 mg/kg for 1^st^ week, 20 mg/kg for 2^nd^ week, 5 times weekly) or EMAP (80 µg/kg, 5 times weekly) for 2 weeks. Animals were euthanized when turning moribund according to predefined criteria including rapid weight loss or gain (>15%), tumor size, lethargy, inability to remain upright and lack of strength. Survival was evaluated from the first day of treatment until death.

### Statistical Analysis

Statistical significance was analyzed by the two-tailed Student’s t-test using GraphPad Prism 4 Software (GraphPad Software, San Diego, CA). In vitro cell proliferation data are expressed as mean ± standard deviation. Additivity of the drug combinations was determined by calculating an “interaction index” using the Chou TC, Talalay P [39] and Lee JJ [40] methods. Statistical analysis for in vivo studies was performed by ANOVA for multiple group comparison and Student’s t-test for the individual group comparison. Survival study statistics were evaluated with StatView for Macintosh (SAS, Carey, NC) by nonparametric survival statistics and logrank testing. Values of p<0.05 were considered to represent statistically significant group differences.

## Results

### Effect of Gemcitabine, Bevacizumab, Sunitinib and EMAP on Cell Proliferation

In vitro cell proliferation analysis in AsPC-1 cells by gemcitabine (10 µM), bevacizumab (1 mg/ml), sunitinib (10 µM) and EMAP (10 µM) showed 35, 22, 81 and 6 percent inhibition in cell proliferation, respectively. Combinations of gemcitabine with single antiangiogenic agents bevacizumab or EMAP had no additive effects, while the combination of gemcitabine with sunitinib could not surpass the effects of single agent sunitinib. Combinations of more than one antiangiogenic agent with gemcitabine had no additive in vitro effects (Table 1). Although gemcitabine had various effects on the other PDAC cell lines BxPC-3, MIA PaCa-2 and Panc-1, the effects of combinations of antiangiogenic agents had similar results as those observed with AsPC-1 (data not shown).

In HUVECs, gemcitabine (10 µM), bevacizumab (1 mg/ml), sunitinib (10 µM) and EMAP (10 µM) treatment caused a 70, 41, 86 and 67 percent inhibition in cell proliferation, respectively. Combinations of gemcitabine with single agent bevacizumab, sunitinib or EMAP resulted in significant additive effects on proliferation inhibition. However, combination of more than one antiangiogenic agent did not have additive effect. In WI-38 cells, gemcitabine (10 µM), bevacizumab (1 mg/ml), sunitinib (10 µM) and EMAP (10 µM) caused 79, 58, 80 and 29 percent inhibition in cell proliferation, respectively. Combination of gemcitabine with bevacizumab, sunitinib or EMAP had significant additive effect and combination of more than one antiangiogenic agents to gemcitabine had no further additive effects (Table 1). For gemcitabine combinations with different antiangiogenic agents, the median interaction index was 1.03 (range 0.9 to 1.34) for AsPC-1 cells, 1.3 (range 1.06 to 2.59) for HUVECs and 1.35 (range 1.06 to 2.47) for WI-38 cells. The interaction indices were obtained at IC_25_, IC_50_, IC_75_ and IC_90_ levels and were not significantly different from 1 indicating that in all drug combinations the combined effects were additive.

### Effects of Gemcitabine, Bevacizumab, Sunitinib and EMAP on Apoptosis Related Proteins

We examined if the inhibition in cell viability by gemcitabine, bevacizumab, sunitinib and EMAP could in part be correlated with induction of apoptosis. Evaluation of PARP-1 cleavage and caspase-3 cleavage as markers of induction in apoptosis revealed that in AsPC-1 cells gemcitabine treatment caused a small increase, bevacizumab and EMAP caused no increase, but sunitinib treatment led to a significant increase. Combination of gemcitabine with sunitinib had additive effects (Figure 1). In HUVECs, gemcitabine, bevacizumab and EMAP caused a small increase and sunitinib caused a strong increase in PARP-1 and caspase-3 cleavage. Combinations of gemcitabine with antiangiogenic agents had additive effects. In WI-38 cells, gemcitabine and sunitinib both caused an obvious increase; while bevacizumab and EMAP demonstrated no significant effect on PARP-1 and caspase-3 cleavage. Combinations of gemcitabine with bevacizumab, sunitinib and EMAP all had additive effects on cleaved PARP-1 and caspase-3 protein expression (Figure 1).

### Effects of Gemcitabine, Bevacizumab, Sunitinib and EMAP on Local Tumor Growth

Subcutaneous murine PDAC xenografts studies showed that gemcitabine, bevacizumab, sunitinib and EMAP treatment inhibited local tumor growth, with sunitinib monotherapy having the strongest effect. Net tumor growth inhibition after a 2-week treatment with gemcitabine, bevacizumab and sunitinib and EMAP was 43, 38, 94 and 46 percent, respectively (Figure 2). Addition of single agent bevacizumab, sunitinib and EMAP to gemcitabine had additive effects on tumor growth inhibition, as Gem+Bev, Gem+Su and Gem+EMAP led to 69, 99 and 64 percent tumor growth inhibition. Combinations of more than one antiangiogenic agent with gemcitabine were also effective but not significantly better in this experiment than sunitinib alone (Figure 2, Figure S1). No apparent signs of drug related toxicity were observed in any treatment group in terms of animal habitus, activity levels and weight (Figure S2).

### Effects of Gemcitabine, Bevacizumab, Sunitinib and EMAP on Intratumoral Proliferative and Apoptotic Activity

Analysis of Ki67-positive cells as marker of intratumoral proliferative activity revealed that gemcitabine, bevacizumab, sunitinib and EMAP monotherapy caused 34, 28, 82 and 22 percent inhibition in the proliferative index. Combinations of gemcitabine with one or more antiangiogenic agents were effective but did not enhance inhibition of the intratumoral proliferative index beyond levels achieved by sunitinib alone (Figure 3).

Evaluation of intratumoral apoptosis by TUNEL-staining demonstrated small increases in apoptosis by gemcitabine, bevacizumab and EMAP, and a significant increase by sunitinib alone. Combinations of bevacizumab and sunitinib or EMAP with gemcitabine led to increased levels of apoptosis compared with single agents. Apoptotic indices (TUNEL-positive cells/total cell per HPF) in controls, and in Gem, Bev, Su, EMAP, Gem+Bev, Gem+Su, Gem+EMAP groups were 0.13±0.03, 0.21±0.06, 0.19±0.03, 0.47±0.05, 0.18±0.01, 0.26±0.03, 0.53±0.01 and 0.25±0.02, respectively. Combinations of more than one antiangiogenic agent with gemcitabine also increased apoptosis, but were not significantly more effective than sunitinib alone (Figure 4).

### Effects of Gemcitabine, Bevacizumab, Sunitinib and EMAP on Intratumoral Microvessel Density

PECAM-1 staining of tumor tissue sections to study tumor vasculature revealed that gemcitabine, bevacizumab, sunitinib and EMAP all caused a significant reduction in microvessel density compared with control (Figure 5). Sunitinib alone induced the maximum effect on decreasing MVD among all agents tested. Combinations of gemcitabine with bevacizumab and EMAP showed additive effects on decreasing microvessel counts compared with single agents. All combinations with sunitinib were very effective, but did not surpass sunitinib alone effects. All groups were evaluated for mean microvessel counts including, controls (25.5±3.5), gemcitabine (14.2±2.1), bevacizumab (11.8±2.6), sunitinib (5.0±2.3), EMAP (11.1±2.5), Gem+Bev (7.8±2), Gem+Su (5.6±1.1), Gem+EMAP (7.9±1.7), Bev+Su (2.9±1), Bev+EMAP (8.2±1.5), Su+EMAP (5.2±1.5), Gem+Bev+Su (3.9±1), Gem+Bev+EMAP (5.5±1.5), Gem+Su+EMAP (3.3±0.6), and Gem+Bev+Su+EMAP (2.9±1), respectively (Figure 5).

### Effects of Gemcitabine, Bevacizumab, Sunitinib and EMAP on Animal Survival

PDAC murine xenograft studies in SCID-NOD mice resulted in a median survival of 19 days in the control group. Median survival (m.s.) increased modestly after gemcitabine (26 days, p = 0.02), but there was no significant survival benefit with single agent bevacizumab, sunitinib or EMAP as compared with control. Combination treatment of gemcitabine with single agent bevacizumab, sunitinib and EMAP all significantly improved animal survival to a similar extent, with median survivals in the Gem+Bev group of 36 days (p = 0.003 vs. control, 0.006 vs. Gem), after Gem+Su 37 days (p = 0.003 vs. control, 0.01 vs. Gem) and after Gem+EMAP 36 days (p = 0.002 vs. control, 0.001 vs. Gem). The combination of gemcitabine with Su+EMAP (m.s. = 36 days) was not better than combination of gemcitabine with single agent sunitinib or EMAP. Combination of gemcitabine with Bev+EMAP (m.s. = 43 days, p = 0.001 vs. control, 0.005 vs. Gem) or with Bev+Su+EMAP (46 days, p = 0.001 vs. control, 0.003 vs. Gem) demonstrated the maximum survival benefit (Figure 6).

## Discussion

Pancreatic cancer has an extremely poor prognosis due to late-stage diagnosis, early metastatic spread and high resistance to radiation and chemotherapy. Although single agent gemcitabine therapy has produced some clinical benefits for metastatic PDAC, the overall survival benefit remains limited [1]. Since angiogenesis is critical for primary and metastatic PDAC progression, antiangiogenic treatment is a sensible and still promising therapeutic avenue due to its potential for synergistic interaction with other antitumor agents, low toxicity and enhanced antitumor effect [41], [42]. Several growth factors such as VEGF, fibroblast growth factor (FGF), PDGF or insulin-like growth factor (IGF) are among the most important angiogenic activators in PDAC progression. Bevacizumab, the first FDA-approved angiogenesis inhibitor showed some promise in initial PDAC studies in combination with gemcitabine but failed to confirm any significant survival benefit in later studies [17]. Sunitinib, a multitargeted inhibitor of angiogenic RTKs VEGFR, PDGFR, c-KIT, FLT-3 and RET, is an interesting agent regarding its combination therapy potential, as compared to narrow-spectrum inhibitors that have so far shown rather limited clinical activity. Our results show that 1.: the combination of antiangiogenic agents with gemcitabine generally yields better results than monotherapy; 2.: the effects of sunitinib alone can be rather pronounced, at least in the models tested; 3.: multiple combinations of antiangiogenic agents in addition to gemcitabine tend to yield the best results; and 4.: sunitinib and bevacizumab appear to have no obvious combination benefit.

Tumor progression critically depends on a complex interaction among several components including tumor cells, immune cells, ECs, extracellular matrix (ECM) and stromal fibroblasts. Solid tumor treatment by targeting the EC and fibroblast compartments within the tumor microenvironment has shown some substantial benefits [43], [44]. EMAP, a tumor-derived antiangiogenic and antiendothelial cytokine, in our experience exhibited antitumor effects in several tumor types including PDAC [28], [30], [31], [34], [35], [45], [46]. The scope of the present study was to evaluate enhancement of gemcitabine response by combination of more than one antiangiogenic agents with an entire spectrum of different mechanisms against PDAC. For instance, we recently demonstrated that EMAP improves gemcitabine plus bevacizumab combination effects against PDAC [34]. Adding a multi-TKR targeting agent such as sunitinib appeared therefore sensible. Gemcitabine had differential growth inhibitory response on PDAC cells in vitro. Gemcitabine sensitivity was seen in the order of BxPC-3> MIA PaCa-2>Panc-1> AsPC-1, indicating BxPC-3 as most sensitive and AsPC-1 as least sensitive cells (Figure S3). Perhaps this is a rather useful approach that allows the testing of additional mechanisms for an in vivo combination benefit in contrast to PDAC lines that are gemcitabine-sensitive. Among antiangiogenic agents, bevacizumab and EMAP alone had no meaningful effect on AsPC-1 proliferation, while sunitinib was very effective. The sunitinib effect was stronger than equimolar gemcitabine, while addition of two or more antiangiogenic agents was not more inhibitory than sunitinib alone. As expected, all three antiangiogenic agents significantly inhibited EC and fibroblast proliferation in vitro, with sunitinib alone again being the most effective. In our studies, since sunitinib had maximum in vitro activity towards tumor cells, ECs and fibroblasts, it supports the assumption that in vivo antitumor activities of sunitinib may partially depend upon its impact on tumor cells as well as tumor vasculature and stromal components, as previously reported [20], . Mendel et al. [20] showed that the sunitinib IC_50_ for VEGF- and PGDF-induced HUVECs was in a nanomolar range. A comparatively weak activity of sunitinib was observed in the present study; we assume that this is due to the use of full-growth medium for HUVECs, which could render cells more resistant to tyrosine kinase inhibitory effects. These results support the notion that benefits can be derived from combinations of multi-targeting agents over agents with limited targets alone, or in addition to them. Gemcitabine and sunitinib have been shown to induce apoptosis in tumor cells as well as endothelial cells [49], [50], [51], [52], whereas bevacizumab and EMAP mainly have proapoptotic activity towards endothelial cells [28], [53]. In the present study, evaluation of induction in apoptosis by gemcitabine and antiangiogenic agents, either alone or in combination, was correlated to cell proliferation results indicating that loss in cell viability by these agents may in part be due to the induction in apoptosis.

In vivo murine xenograft studies demonstrated that gemcitabine, bevacizumab, sunitinib and EMAP inhibited local tumor growth as single agent. Antitumor effects of bevacizumab and EMAP are more likely due to their effect on ECs and fibroblasts rather than tumor cells, and accordingly, the impact of these agents as monotherapy was limited. Sunitinib as single agent was very effective, likely due to its broad target spectrum, inhibiting multiple cellular compartments in the tumor microenvironment. Despite using less than half the maximum tolerable dose of sunitinib its high effect could have masked any combination treatment benefits. A lower dose of sunitinib in the combination group would have provided more useful evaluation of the effect of combination treatment. Gemcitabine effects on tumor growth inhibition were enhanced by the addition of single antiangiogenic agents, generally supporting the importance of blocking multiple pathways for the more effective treatment of PDAC. Tumor growth inhibition results in the present study appear to be correlated with intratumoral proliferative index, apoptotic index and microvessel density. However, a predictive factor suggestive of this specific treatment response cannot be determined due to lack of a specific mechanism of action and the use of only a single PDAC cell line. Several distinct mechanisms including induction of cancer cell proliferation, migration, differentiation, adhesion, angiogenesis and inhibition of cancer or stromal cell apoptosis are responsible for active PDAC progression. Gemcitabine has antiproliferative and proapoptotic effects on tumor cells as well as ECs and fibroblasts. The exact operational mechanisms for the enhancement in antitumor activity of gemcitabine in combination with bevacizumab, sunitinib or EMAP remain unclear; however, they more likely include normalization of tumor microvessels, increased delivery of gemcitabine into the tumor tissue by reducing interstitial pressure, prevention of rapid tumor cell repopulation during successive gemcitabine courses, and reduction of stromal mechanisms for tumor cell progression; less likely, augmentation of direct antitumor effects of gemcitabine is suspected [54], [55], [56].

As expected, bevacizumab and EMAP as single agent did not improve animal survival; interestingly, and in contrast to the in vitro proliferation and local tumor growth inhibition data, sunitinib alone did not improve survival either. This might be due to an increased metastatic burden in this intraperitoneal xenograft model of PDAC, or possibly mechanisms of intraperitoneal tumor progression that are different from local subcutaneous settings. Importantly, gemcitabine based survival effects were significantly enhanced by addition of single agent bevacizumab, sunitinib or EMAP, and combinations of gemcitabine with dual agent Bev+EMAP or triple agent Bev+Su+EMAP were even most effective. Based on the negative clinical trial results of gemcitabine and bevacizumab in combination [17], our study clearly demonstrates the need for testing combinations of gemcitabine with other, mechanistically different antiangiogenic agents, especially multitargeted agents such as sunitinib.

In summary, the present study demonstrates that certain combinations of antiangiogenic agents with gemcitabine improve in vivo antitumor effects, in accordance with differential inhibitory effects on various cell types in vitro. Among the three antiangiogenic agents tested, sunitinib was rather effective either alone or in combination with gemcitabine. These results strongly corroborate the benefits of combining polymechanistic, multi-targeting antiangiogenic agents with standard gemcitabine therapy for clinical PDAC treatment. In addition, these studies indicate benefits of selecting antiangiogenic combinations based on their mechanistic compatibility compared with random or non-selective combinations.

## Supporting Information

Figure S1 **Effects of gemcitabine (Gem), bevacizumab (Bev), sunitinib (Su) and EMAP therapy on local tumor growth.** Nude mice were subcutaneously injected with AsPC-1 cell (0.75×10^6^). Fourteen days after tumor cell injection, therapy was started with Gem, Bev, Su and EMAP for 2 weeks. Tumor growth was measured twice a week using calipers. Relative tumor volume was calculated by dividing the tumor volume at any time by the tumor volume at the start of therapy. Data are representative of mean values ± standard deviation from 6–8 mice per group.
(TIF)

Figure S2 **Effects of gemcitabine (Gem), bevacizumab (Bev), sunitinib (Su) and EMAP therapy on mouse body weight.** Nude mice were subcutaneously injected with AsPC-1 cell (0.75×10^6^). Fourteen days after tumor cell injection, therapy was started with Gem, Bev, Su and EMAP for 2 weeks. Mouse body weight was measured twice a week. Data are representative of mean values ± standard deviation from 6–8 mice per group.
(TIF)

Figure S3 **Effect of gemcitabine on in vitro cell proliferation of PDAC cells.** AsPC-1, BxPC-3, MIA PaCa-2 and Panc-1 cells were plated on 96-well plate and treated with 100 nM, 500 nM, 1 µM and 10 µM concentrations of gemcitabine. After 72 h, 10 µl WST-1 reagent was added in each well and incubated for 2 additional hours. The absorbance at 450 nm was measured using microplate reader. The resulting number of viable cells was calculated by measuring absorbance of color produced in each well. Data are the mean ± standard deviation of triplicate determinations.
(TIF)

## References

[1] Jemal A, Siegel R, Xu J, Ward E (2010). Cancer statistics, 2010.. CA Cancer J Clin.

[2] Brennan MF (2004). Adjuvant therapy following resection for pancreatic adenocarcinoma.. Surg Oncol Clin N Am 13: 555–566, vii.

[3] Rosewicz S, Wiedenmann B (1997). Pancreatic carcinoma.. Lancet.

[4] Wilkowski R, Thoma M, Bruns C, Duhmke E, Heinemann V (2006). Combined chemoradiotherapy for isolated local recurrence after primary resection of pancreatic cancer.. JOP.

[5] Reni M, Cordio S, Milandri C, Passoni P, Bonetto E (2005). Gemcitabine versus cisplatin, epirubicin, fluorouracil, and gemcitabine in advanced pancreatic cancer: a randomised controlled multicentre phase III trial.. Lancet Oncol.

[6] Rocha Lima CM, Green MR, Rotche R, Miller WH, Jeffrey GM (2004). Irinotecan plus gemcitabine results in no survival advantage compared with gemcitabine monotherapy in patients with locally advanced or metastatic pancreatic cancer despite increased tumor response rate.. J Clin Oncol.

[7] Louvet C, Labianca R, Hammel P, Lledo G, Zampino MG (2005). Gemcitabine in combination with oxaliplatin compared with gemcitabine alone in locally advanced or metastatic pancreatic cancer: results of a GERCOR and GISCAD phase III trial.. J Clin Oncol.

[8] Garcea G, Lloyd TD, Gescher A, Dennison AR, Steward WP (2004). Angiogenesis of gastrointestinal tumours and their metastases–a target for intervention?. Eur J Cancer.

[9] Ko AH, Dito E, Schillinger B, Venook AP, Xu Z (2008). A phase II study evaluating bevacizumab in combination with fixed-dose rate gemcitabine and low-dose cisplatin for metastatic pancreatic cancer: is an anti-VEGF strategy still applicable?. Invest New Drugs.

[10] Dragovich T, Burris H, Loehrer P, Von Hoff DD, Chow S (2008). Gemcitabine plus celecoxib in patients with advanced or metastatic pancreatic adenocarcinoma: results of a phase II trial.. Am J Clin Oncol.

[11] Bramhall SR, Schulz J, Nemunaitis J, Brown PD, Baillet M (2002). A double-blind placebo-controlled, randomised study comparing gemcitabine and marimastat with gemcitabine and placebo as first line therapy in patients with advanced pancreatic cancer.. Br J Cancer.

[12] Moore MJ, Goldstein D, Hamm J, Figer A, Hecht JR (2007). Erlotinib plus gemcitabine compared with gemcitabine alone in patients with advanced pancreatic cancer: a phase III trial of the National Cancer Institute of Canada Clinical Trials Group.. J Clin Oncol.

[13] Korc M (2003). Pathways for aberrant angiogenesis in pancreatic cancer.. Mol Cancer.

[14] Luo J, Guo P, Matsuda K, Truong N, Lee A (2001). Pancreatic cancer cell-derived vascular endothelial growth factor is biologically active in vitro and enhances tumorigenicity in vivo.. Int J Cancer.

[15] Itakura J, Ishiwata T, Shen B, Kornmann M, Korc M (2000). Concomitant over-expression of vascular endothelial growth factor and its receptors in pancreatic cancer.. Int J Cancer.

[16] Ogawa T, Takayama K, Takakura N, Kitano S, Ueno H (2002). Anti-tumor angiogenesis therapy using soluble receptors: enhanced inhibition of tumor growth when soluble fibroblast growth factor receptor-1 is used with soluble vascular endothelial growth factor receptor.. Cancer Gene Ther.

[17] Cabebe E, Fisher GA (2007). Clinical trials of VEGF receptor tyrosine kinase inhibitors in pancreatic cancer.. Expert Opin Investig Drugs.

[18] Abrams TJ, Lee LB, Murray LJ, Pryer NK, Cherrington JM (2003). SU11248 inhibits KIT and platelet-derived growth factor receptor beta in preclinical models of human small cell lung cancer.. Mol Cancer Ther.

[19] O’Farrell AM, Abrams TJ, Yuen HA, Ngai TJ, Louie SG (2003). SU11248 is a novel FLT3 tyrosine kinase inhibitor with potent activity in vitro and in vivo.. Blood.

[20] Mendel DB, Laird AD, Xin X, Louie SG, Christensen JG (2003). In vivo antitumor activity of SU11248, a novel tyrosine kinase inhibitor targeting vascular endothelial growth factor and platelet-derived growth factor receptors: determination of a pharmacokinetic/pharmacodynamic relationship.. Clin Cancer Res.

[21] Abrams TJ, Murray LJ, Pesenti E, Holway VW, Colombo T (2003). Preclinical evaluation of the tyrosine kinase inhibitor SU11248 as a single agent and in combination with “standard of care” therapeutic agents for the treatment of breast cancer.. Mol Cancer Ther.

[22] Fujimoto K, Hosotani R, Wada M, Lee JU, Koshiba T (1998). Expression of two angiogenic factors, vascular endothelial growth factor and platelet-derived endothelial cell growth factor in human pancreatic cancer, and its relationship to angiogenesis.. Eur J Cancer.

[23] Korc M (2007). Pancreatic cancer-associated stroma production.. Am J Surg.

[24] Chang YT, Chang MC, Wei SC, Tien YW, Hsu C (2008). Serum vascular endothelial growth factor/soluble vascular endothelial growth factor receptor 1 ratio is an independent prognostic marker in pancreatic cancer.. Pancreas.

[25] Cuneo KC, Geng L, Fu A, Orton D, Hallahan DE (2008). SU11248 (sunitinib) sensitizes pancreatic cancer to the cytotoxic effects of ionizing radiation.. Int J Radiat Oncol Biol Phys.

[26] Tran Cao HS, Bouvet M, Kaushal S, Keleman A, Romney E (2010). Metronomic gemcitabine in combination with sunitinib inhibits multisite metastasis and increases survival in an orthotopic model of pancreatic cancer.. Mol Cancer Ther.

[27] Awasthi N, Schwarz MA, Schwarz RE (2011). Antitumour activity of sunitinib in combination with gemcitabine in experimental pancreatic cancer.. HPB (Oxford).

[28] Schwarz MA, Kandel J, Brett J, Li J, Hayward J (1999). Endothelial-monocyte activating polypeptide II, a novel antitumor cytokine that suppresses primary and metastatic tumor growth and induces apoptosis in growing endothelial cells.. J Exp Med.

[29] Berger AC, Alexander HR, Tang G, Wu PS, Hewitt SM (2000). Endothelial monocyte activating polypeptide II induces endothelial cell apoptosis and may inhibit tumor angiogenesis.. Microvasc Res.

[30] Schwarz RE, Awasthi N, Konduri S, Caldwell L, Cafasso D (2010). Antitumor effects of EMAP II against pancreatic cancer through inhibition of fibronectin-dependent proliferation.. Cancer Biol Ther.

[31] Schwarz RE, Schwarz MA (2004). In vivo therapy of local tumor progression by targeting vascular endothelium with EMAP-II.. J Surg Res.

[32] Awasthi N, Schwarz MA, Verma V, Cappiello C, Schwarz RE (2009). Endothelial monocyte activating polypeptide II interferes with VEGF-induced proangiogenic signaling.. Lab Invest.

[33] Schwarz MA, Zheng H, Liu J, Corbett S, Schwarz RE (2005). Endothelial-monocyte activating polypeptide II alters fibronectin based endothelial cell adhesion and matrix assembly via alpha5 beta1 integrin.. Exp Cell Res.

[34] Schwarz RE, Awasthi N, Konduri S, Cafasso D, Schwarz MA (2010). EMAP II-based antiangiogenic-antiendothelial in vivo combination therapy of pancreatic cancer.. Ann Surg Oncol.

[35] Schwarz RE, Konduri S, Awasthi N, Cafasso D, Schwarz MA (2009). An antiendothelial combination therapy strategy to increase survival in experimental pancreatic cancer.. Surgery.

[36] Awasthi N, Schwarz MA, Schwarz RE (2011). Enhancing cytotoxic agent activity in experimental pancreatic cancer through EMAP II combination therapy.. Cancer Chemother Pharmacol.

[37] Schwarz MA, Zhang F, Gebb S, Starnes V, Warburton D (2000). Endothelial monocyte activating polypeptide II inhibits lung neovascularization and airway epithelial morphogenesis.. Mech Dev.

[38] Schwarz RE, McCarty TM, Peralta EA, Diamond DJ, Ellenhorn JD (1999). An orthotopic in vivo model of human pancreatic cancer.. Surgery.

[39] Chou TC, Talalay P (1984). Quantitative analysis of dose-effect relationships: the combined effects of multiple drugs or enzyme inhibitors.. Adv Enzyme Regul.

[40] Lee JJ, Kong M, Ayers GD, Lotan R (2007). Interaction index and different methods for determining drug interaction in combination therapy.. J Biopharm Stat.

[41] Konno H, Tanaka T, Matsuda I, Kanai T, Maruo Y (1995). Comparison of the inhibitory effect of the angiogenesis inhibitor, TNP-470, and mitomycin C on the growth and liver metastasis of human colon cancer.. Int J Cancer.

[42] Satoh H, Ishikawa H, Fujimoto M, Fujiwara M, Yamashita YT (1998). Angiocytotoxic therapy in human non-small cell lung cancer cell lines–advantage of combined effects of TNP-470 and SN-38.. Acta Oncol.

[43] Hayes AJ, Li LY, Lippman ME (2000). Anti-vascular therapy: a new approach to cancer treatment.. West J Med.

[44] Kalluri R, Zeisberg M (2006). Fibroblasts in cancer.. Nat Rev Cancer.

[45] Reznikov AG, Chaykovskaya LV, Polyakova LI, Kornelyuk AI (2007). Antitumor effect of endothelial monocyte-activating polypeptide-II on human prostate adenocarcinoma in mouse xenograft model.. Exp Oncol.

[46] Crippa L, Gasparri A, Sacchi A, Ferrero E, Curnis F (2008). Synergistic damage of tumor vessels with ultra low-dose endothelial-monocyte activating polypeptide-II and neovasculature-targeted tumor necrosis factor-alpha.. Cancer Res.

[47] Chow LQ, Eckhardt SG (2007). Sunitinib: from rational design to clinical efficacy.. J Clin Oncol.

[48] Seandel M, Shia J, Linkov I, Maki RG, Antonescu CR (2006). The activity of sunitinib against gastrointestinal stromal tumor seems to be distinct from its antiangiogenic effects.. Clin Cancer Res.

[49] Bold RJ, Chandra J, McConkey DJ (1999). Gemcitabine-induced programmed cell death (apoptosis) of human pancreatic carcinoma is determined by Bcl-2 content.. Ann Surg Oncol.

[50] Laquente B, Lacasa C, Ginesta MM, Casanovas O, Figueras A (2008). Antiangiogenic effect of gemcitabine following metronomic administration in a pancreas cancer model.. Mol Cancer Ther.

[51] Yang F, Jove V, Xin H, Hedvat M, Van Meter TE (2010). Sunitinib induces apoptosis and growth arrest of medulloblastoma tumor cells by inhibiting STAT3 and AKT signaling pathways.. Mol Cancer Res.

[52] Zhang HP, Takayama K, Su B, Jiao XD, Li R (2011). Effect of sunitinib combined with ionizing radiation on endothelial cells.. J Radiat Res (Tokyo).

[53] Costa R, Carneiro A, Rocha A, Pirraco A, Falcao M (2009). Bevacizumab and ranibizumab on microvascular endothelial cells: A comparative study.. J Cell Biochem.

[54] Jain RK (2005). Normalization of tumor vasculature: an emerging concept in antiangiogenic therapy.. Science.

[55] Kerbel RS (2006). Antiangiogenic therapy: a universal chemosensitization strategy for cancer?. Science.

[56] Jain RK, Duda DG, Clark JW, Loeffler JS (2006). Lessons from phase III clinical trials on anti-VEGF therapy for cancer.. Nat Clin Pract Oncol.

