# Supplementary figures and images for: Evaluation of Poly-Mechanistic Antiangiogenic Combinations to Enhance Cytotoxic Therapy Response in Pancreatic Cancer
Source: PLoS One. 2012 Jun 18;7(6):e38477. doi: 10.1371/journal.pone.0038477 (PMC3377661; doi:10.1371/journal.pone.0038477)

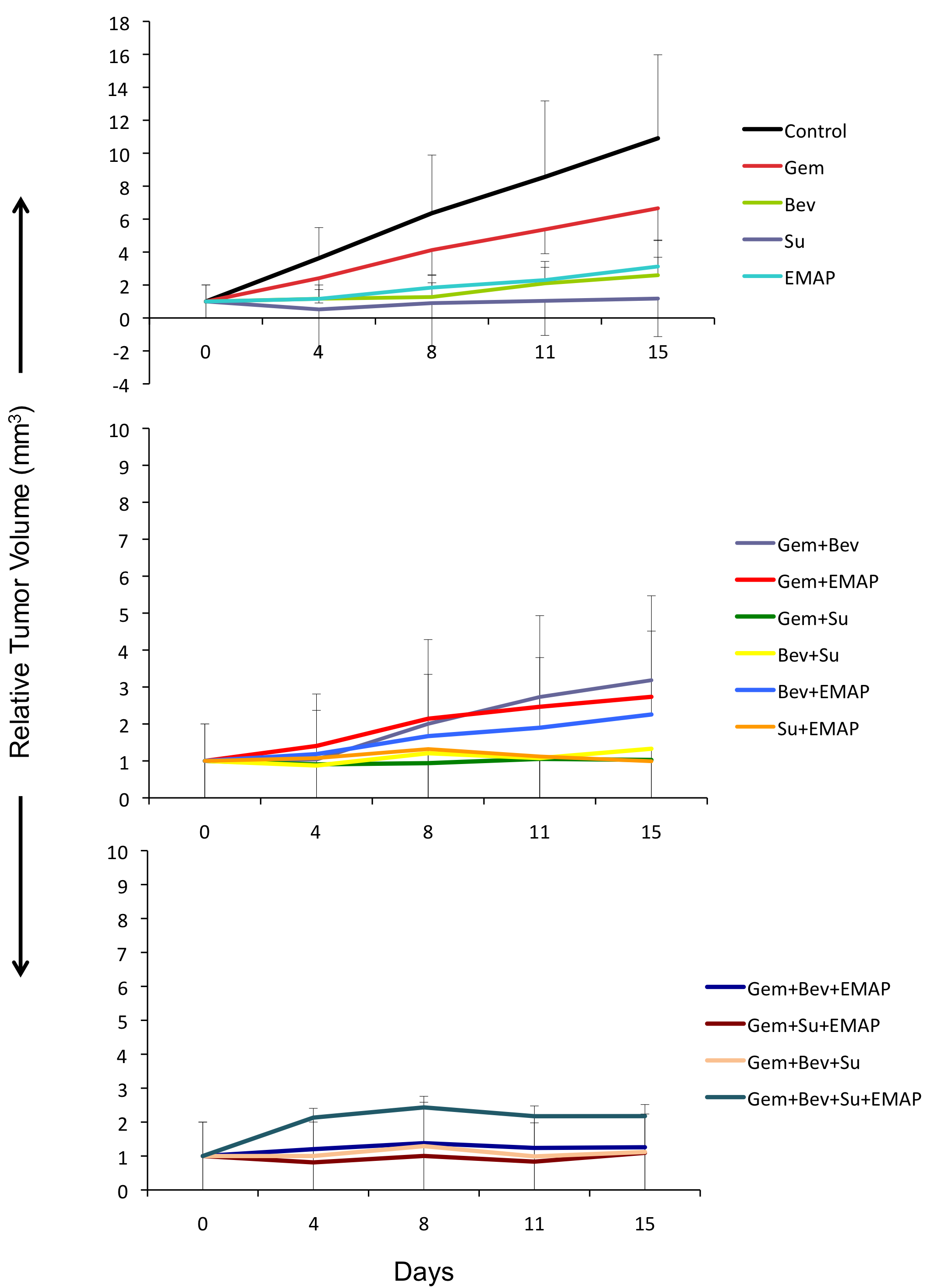

Supplement: Figure S1 — Effects of gemcitabine (Gem), bevacizumab (Bev), sunitinib (Su) and EMAP therapy on local tumor growth. Nude mice were subcutaneously injected with AsPC-1 cell (0.75×10^6^). Fourteen days after tumor cell injection, therapy was started with Gem, Bev, Su and EMAP for 2 weeks. Tumor growth was measured twice a week using calipers. Relative tumor volume was calculated by dividing the tumor volume at any time by the tumor volume at the start of therapy. Data are representative of mean values ± standard deviation from 6–8 mice per group. (TIF) [file pone.0038477.s001.tif]

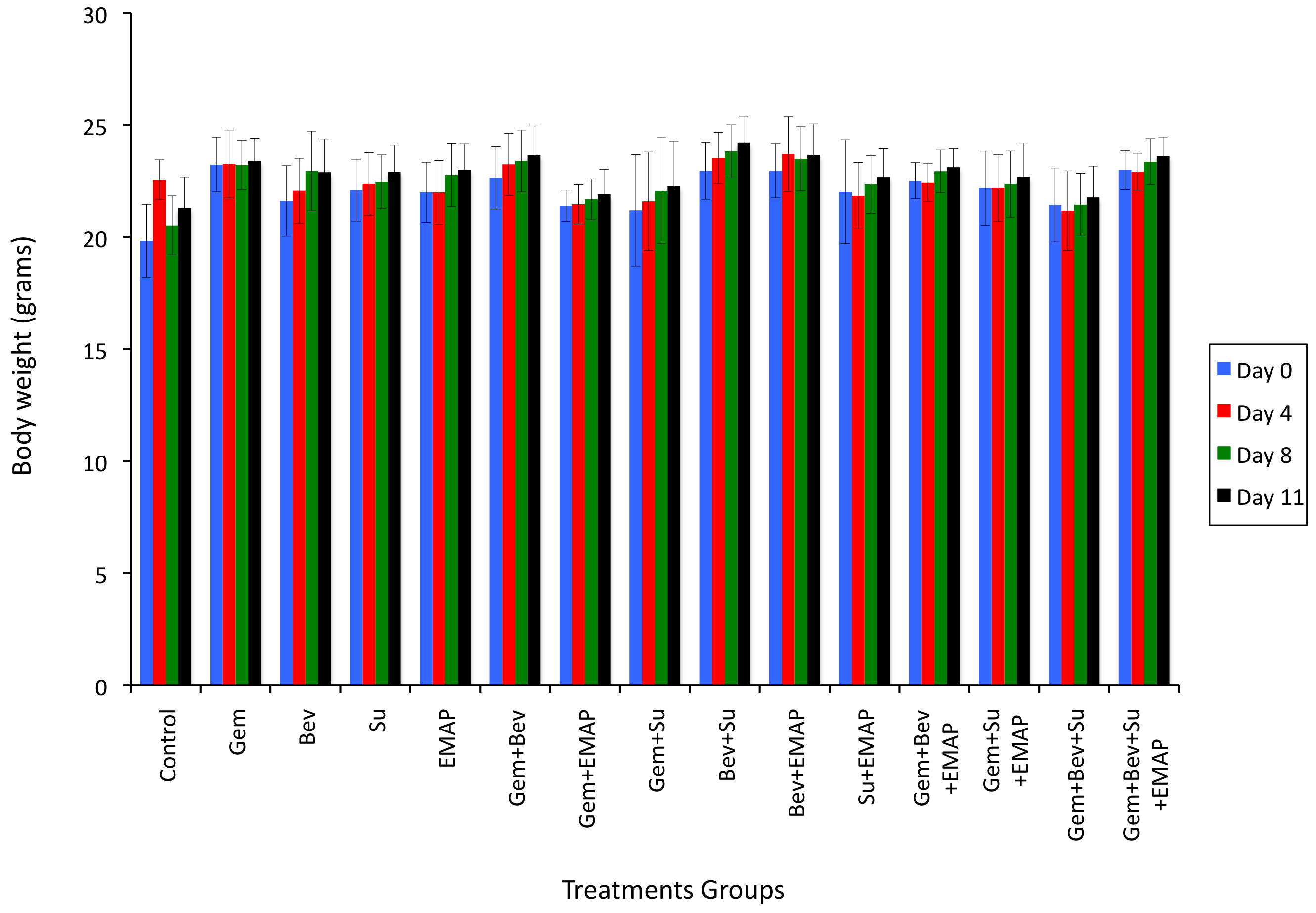

Supplement: Figure S2 — Effects of gemcitabine (Gem), bevacizumab (Bev), sunitinib (Su) and EMAP therapy on mouse body weight. Nude mice were subcutaneously injected with AsPC-1 cell (0.75×10^6^). Fourteen days after tumor cell injection, therapy was started with Gem, Bev, Su and EMAP for 2 weeks. Mouse body weight was measured twice a week. Data are representative of mean values ± standard deviation from 6–8 mice per group. (TIF) [file pone.0038477.s002.tif]

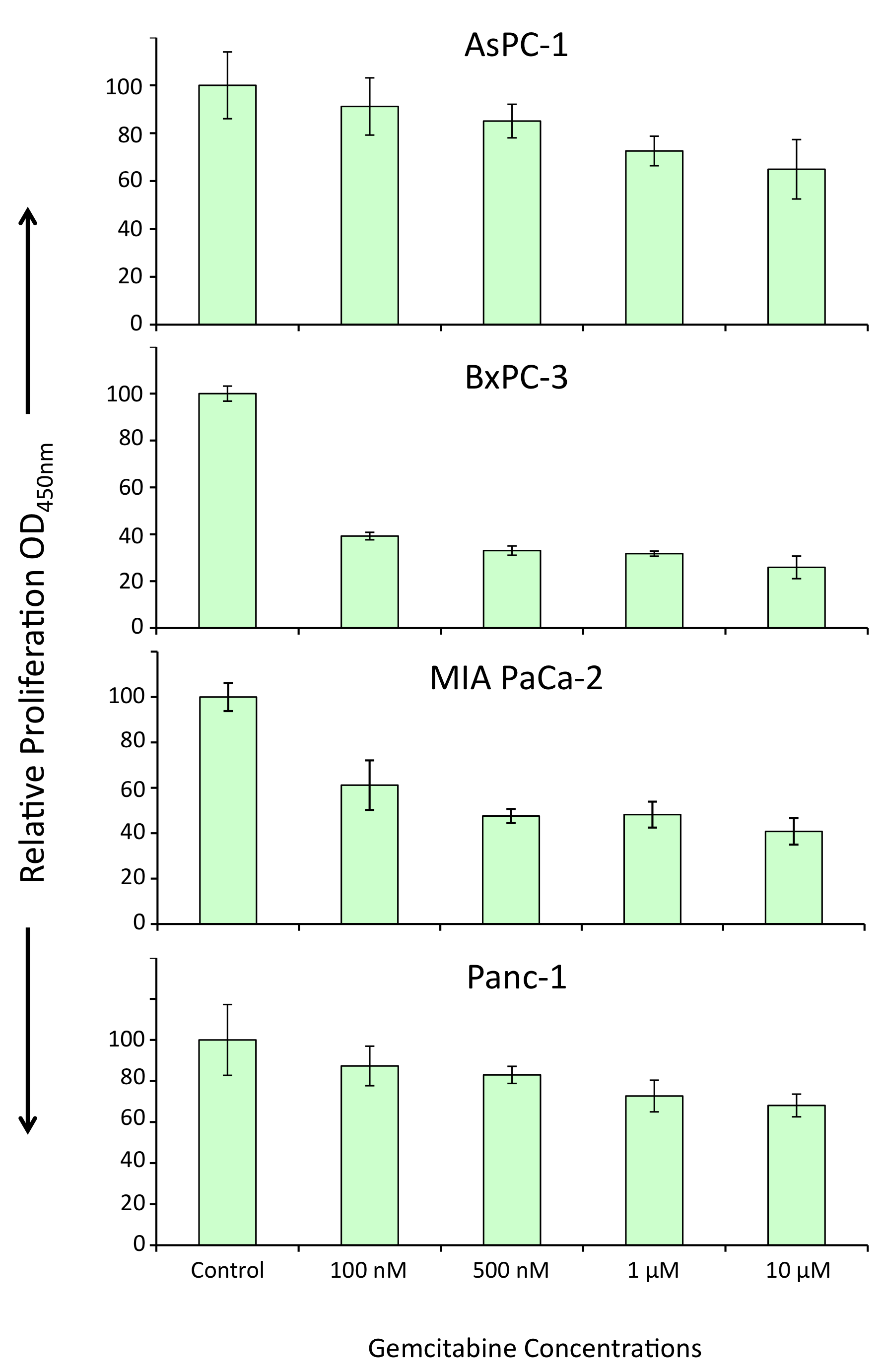

Supplement: Figure S3 — Effect of gemcitabine on in vitro cell proliferation of PDAC cells. AsPC-1, BxPC-3, MIA PaCa-2 and Panc-1 cells were plated on 96-well plate and treated with 100 nM, 500 nM, 1 µM and 10 µM concentrations of gemcitabine. After 72 h, 10 µl WST-1 reagent was added in each well and incubated for 2 additional hours. The absorbance at 450 nm was measured using microplate reader. The resulting number of viable cells was calculated by measuring absorbance of color produced in each well. Data are the mean ± standard deviation of triplicate determinations. (TIF) [file pone.0038477.s003.tif]
